# Evaluation of pooled sample analysis strategy in expediting case detection in areas with emerging outbreaks of COVID-19: A pilot study

**DOI:** 10.1371/journal.pone.0239492

**Published:** 2020-09-22

**Authors:** Anirudh K. Singh, Ram Kumar Nema, Ankur Joshi, Prem Shankar, Shashwati Nema, Arun Raghuwanshi, Chitra Patankar, Bijina J. Mathew, Arti Shrivas, Ritu Pandey, Ranu Tripathi, Debasis Biswas, Sarman Singh

**Affiliations:** 1 Department of Microbiology, All India Institute of Medical Sciences, Bhopal, Madhya Pradesh, India; 2 Regional Virology Laboratory, All India Institute of Medical Sciences, Bhopal, Madhya Pradesh, India; 3 Department of Community and Family Medicine, All India Institute of Medical Sciences, Bhopal, Madhya Pradesh, India; 4 Department of Biochemistry, All India Institute of Medical Sciences, Bhopal, Madhya Pradesh, India; 5 Department of Pediatrics, All India Institute of Medical Sciences, Bhopal, Madhya Pradesh, India; CEA, FRANCE

## Abstract

Timely diagnosis of COVID-19 infected individuals and their prompt isolation are essential for controlling the transmission of SARS-CoV-2. Though quantitative reverse transcriptase PCR (qRT-PCR) is the method of choice for COVID-19 diagnostics, the resource-intensive and time-consuming nature of the technique impairs its wide applicability in resource-constrained settings and calls for novel strategies to meet the ever-growing demand for more testing. In this context, a pooled sample testing strategy was evaluated in the setting of emerging disease outbreak in 3 central Indian districts to assess if the cost of the test and turn-around time could be reduced without compromising its diagnostic characteristics and thus lead to early containment of the outbreak. From 545 nasopharyngeal and oropharyngeal samples received from the three emerging districts, a total of 109 pools were created with 5 consecutive samples in each pool. The diagnostic performance of qRT-PCR on pooled sample was compared with that of individual samples in a blinded manner. While pooling reduced the cost of diagnosis by 68% and the laboratory processing time by 66%, 5 of the 109 pools showed discordant results when compared with induvial samples. Four pools which tested negative contained 1 positive sample and 1 pool which was positive did not show any positive sample on deconvolution. Presence of a single infected sample with Ct value of 34 or higher, in a pool of 5, was likely to be missed in pooled sample analysis. At the reported point prevalence of 4.8% in this study, the negative predictive value of qRT-PCR on pooled samples was around 96% suggesting that the adoption of this strategy as an effective screening tool for COVID-19 needs to be carefully evaluated.

## Introduction

The transmission of COVID-19 is difficult to contain owing to the dual factors of high transmissibility of SARS-CoV-2 (R0 = 2.1–3.3) and asymptomatic/ mildly symptomatic individuals serving as sources of infection [1–4]. Extensive testing of suspected cases and asymptomatic direct and high-risk contacts is, therefore, recommended as key to controlling the ongoing pandemic [5]. However, the testing modality of choice *viz*. quantitative reverse transcriptase PCR (qRT-PCR), is too technically demanding, time-consuming and resource-intensive to be widely adaptable in low- and middle-income countries as well as remote locations and thus it often fails to inform early identification and quick isolation of the infected patients. Innovative methods for expediting the qRT-PCR results, without compromising the diagnostic sensitivity and specificity, are therefore urgently needed.

**Fig 1:**
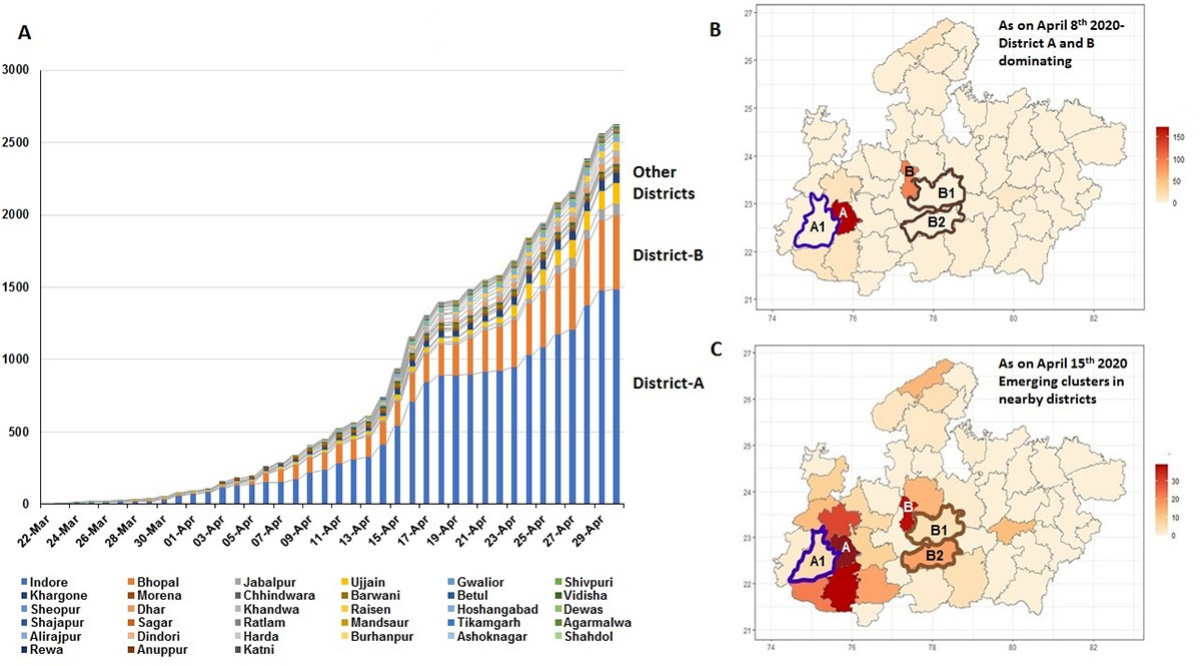
COVID-19 time trends in districts of Madhya Pradesh. **A)** Composite bar charts showing proportional distribution of COVID-19 cases in districts of Madhya Pradesh where district A (Indore) and district B (Bhopal) were contributing to majority of cases with emergence of other clusters in the month of April. **B)** Choropleth map for case density of Madhya Pradesh showing district A and B in darker shades as on 8^th^ April 2020. **C)** Choropleth map for case density of Madhya Pradesh after removing polygons for district A and B (white shades) as on 15^th^ April 2020 (emergence of cases in the adjacent districts are reflected by a darker shades). Pooling strategy was evaluated for the emerging clusters, neighboring district A [A1 (Dhar) blue skeleton cluster] and district B [B1 (Raisen), B2 (Hoshangabad) brown skeleton clusters].

The central Indian state of Madhya Pradesh (MP), with a total of 52 districts, reported the first case of COVID-19 on March 20^th^ 2020 and till 8^th^ April 2020 majority of the reported cases from the state (78.2%) were restricted to the 2 districts of Indore (District A) and Bhopal (District B). From the second week of April 2020, cases started being reported from district Dhar (A1), which borders District A, and from districts Raisen (B1) and Hoshangabad (B2), both of which are adjacent to District B (Fig 1). This prompted massive contact tracing in each of these 3 districts in an effort to contain the spread of infection.

Since the samples collected from these emerging districts converged on a laboratory system that was already over-burdened with unprecedented workload, a need was felt to evaluate strategies for testing additional samples without compromising the diagnostic characteristics and turn-around time of the test.

We hypothesized that testing of pooled respiratory samples, collected from potentially infected individuals, could lead to faster laboratory confirmation and quicker containment of the emerging infection in these districts and, thus, undertook this study to evaluate the diagnostic concordance between the strategies of pooled vs. individualized testing and estimate the gain in turn-around time (TAT) and resources that could be achieved through pooling.

## Material and methods

This study was approved by the All India Institute of Medical Sciences, Bhopal Institutional Human Ethics Committee with the waiver of consent as per the “National Ethical Guidelines for Biomedical and Health Research” set forth by Indian Council of Medical Research, which is an apex government body responsible for making and enforcing policies of medical research in India. As per the guidelines, the institutional ethics committee can grant waiver of consent if the research is done on anonymized biological samples and/or the primary purpose of the research is refinement and improvement of the public health programs. As our study met both these criteria it was approved with the waiver of consent. Nasopharyngeal and oropharyngeal swabs were collected by trained healthcare workers from suspected COVID-19 patients belonging to the districts A1, B1 and B2 in vials containing Viral Transport Medium (VTM) during April and May 2020. Samples were transported at 2–8°C to the testing laboratory within 24 hours. The relevant clinical and epidemiological details of the patients were entered in a standard form approved by the Indian Council of Medical Research, which is spearheading the nationwide laboratory network for COVID-19 testing. Aliquots of the original consecutive clinical samples were anonymized and processed in parallel for individualized and pooled analysis in a blinded manner. A pool size of 5 was chosen as per the advisory of the Indian Council of Medical Research for pooled sample testing for COVID-19. For pooled analysis, 200 μl from each of 5 consecutive samples were collected in a single 1.5 ml centrifuge tube and processed for RNA extraction using QIAamp Viral RNA Mini kit (Qiagen, Hilden, Germany) as per manufacturer’s instructions. RNA extraction for individualized testing was also performed using the same kit. The extracted RNA samples were subjected to diagnosis using Real-Time Fluorescent RT-PCR Kit for Detecting SARS-CoV-2 (BGI, Hong Kong) as per the manufacturer’s protocol on a BioRad CFX96 thermal cycler. The kit targets ORF1ab for detection of the virus and human β-actin as the internal control. As recommended by the manufacturer, a sigmoidal curve with a Ct value ≤ 35 was considered as the criterion for considering a sample as positive for SARS-CoV-2. All the necessary controls namely no-template control, extraction control and positive control were tested in parallel with every batch of samples, as part of the quality control of the procedure.

### Statistical analysis

The data was entered in MS Excel in wide data format. It was checked for missing values, redundancies and outliers. The descriptive summarization of variables of interest was done by median and IQR for ordinal and interval data (non-parametric distribution) and counts for nominal data. The diagnostic accuracy of pooled strategy, in reference to individual qRT-PCR, for correctly classifying the pool was determined by point and interval estimates of sensitivity, specificity and by likelihood ratios. As positive predictive value (PPV) and negative predictive value (NPV) are prevalence-dependent parameters, we ran a simulation to estimate the PPV and NPV values with a varying pre-test probability from 1%-5% in addition to group prevalence. Further a kappa statistic was calculated between the two strategies in order to detect the agreement beyond chance. We also calculated the nonparametric spearman correlation to check the magnitude and direction of the relationship between pooled Ct values and number of positive samples in the same pool. All the analyses were done by base R software which is in open domain and associated epi-R package. The choropleth map was drawn with the aid of gg plot2 package in R software. The Bland Altman (BA) plot was drawn and the relevant BA statistic was calculated with 'Bland Altman Leh' and 'ggplot2' r package.

## Results

A total of 545 samples were collected, with 140, 270 and 135 of them belonging to districts A1, B1 and B2, respectively. Both, the individual samples and their pools were processed in parallel for testing. A total of 109 pools were created from 5 consecutive samples received from each district and the diagnostic performance of the 2 strategies were compared. Considering the individualized qRT-PCR technique as the gold standard, we observed PCR-positivity of 7.1% (10/140), 3.7% (10/270) and 4.4% (6/135) in the districts A1, B1 and B2 respectively.

**Fig 2:**
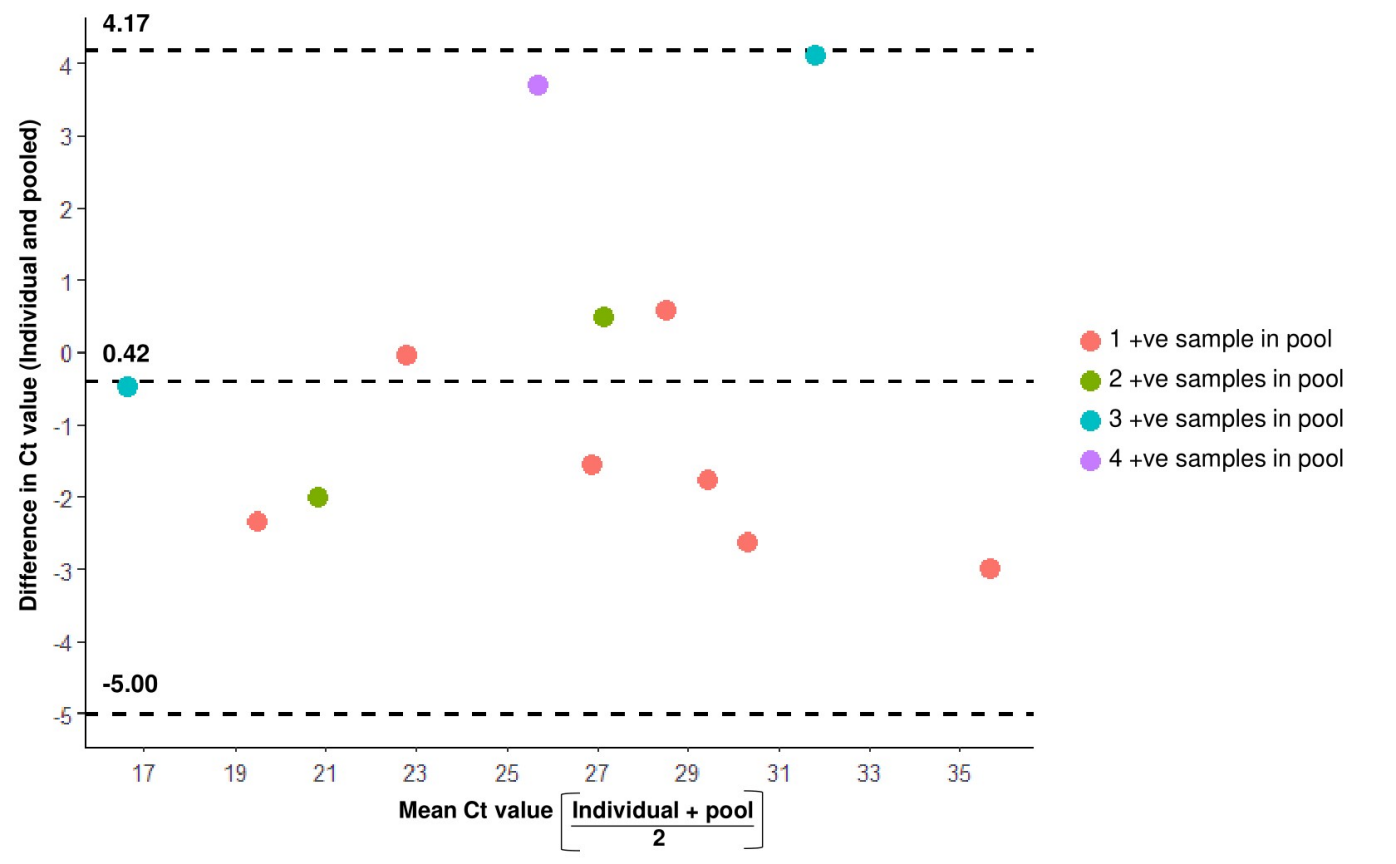
Bland Altman (BA) plot showing the relationship between Ct values of pooled versus individual samples. BA plot was drawn to visualize the relationship between the individual Ct values of sample(s) in a pool with the mean Ct value of the positive sample(s) in the pool. BA lines (dashed lines) assign the mathematical expression of this discrepancy by obtaining the 95% CI of deviation from point estimate (0.42).

The 25 samples that tested positive on individualized testing got sorted into 16 pools and 12 of these 16 pools were detected as positive during pooled sample testing. Majority of these 12 pools contained 1 positive sample (n = 7). While all 5 pools containing more than one positive sample could be detected on pooling, 4 pools each containing single positive sample were missed by this strategy. Although all the pools with more than one positive sample tested positive, we observed a weak negative and non-significant relationship between the pool Ct value and the number of positive samples in the pool by Spearman’s rank correlation test (ρ = -0.39, p = 0.21). The BA plots were computed to further examine the relationship between individual and mean Ct value of positive samples in the pool with reference to number of individual positive samples in pool. It is expected that as the number of positive samples in a pool increases the difference in individual and pooled Ct value should regress to point estimate which is not the case in our analyses. The BA statistic for the line is 0.42 (95% CI: -4.2 to 5.0), which reflects a wide and variegated difference. This difference was not dependent on the proportion of positive samples in the pool, as pools with multiple positive samples were as dispersed and eccentric around mean (or line of no difference towards zero) as positive pools with single positive samples (Fig 2). Four pools, each having one positive sample among their constituents, gave false negative result on pooled analysis. The Ct values of the 4 individual positive samples in these discordant pools were 28.6, 34.0, 34.3 and 34.9 respectively. Comparing these results with the 7 pools that tested positive despite having a single positive sample, we found the Ct values of the samples in the concordant pools to be remarkably lower than the same in the discordant pools. The statistical significance of this difference could not however be determined by Mann Whitney U test, due to the statistical constraint of having less than 5 observations in the discordant group (Table 1).

**Table 1:** Distribution of Ct values on pooled analysis of samples that tested positive on individualized testing.

No. of positive samples in each pool	POSITIVE POOLS			NEGATIVE POOLS	
	No. of positive pools	Median (IQR) Ct value of pool	Median (IQR) Ct value of positive samples on individualized testing	No. of negative pools	Median (IQR) Ct value of positive samples of negative pool on individualized testing
1	7	27.7 (22.6–28.8)	26.1 (20.7–29.4)	4	34.1 (32.7–34.3)
2	2	28.	26.6 (25.6–27.6)	0	-
3*	2	22.7	23.7 (16.6–34.4)	0	-
4	1	27.6	28.6 (26.7–29.8)	0	-
5	0	-	-	0	-

*Note: This category had wide variation in Ct values of 2 pools.

Pool 1: Pool Ct = 16.9 (Individual Ct values = 15.0, 16.1, 18.1)

Pool 2: Pool Ct = 29.2 (Individual Ct values = 29.2, 36.1, 36.3)

For individual and pooled Ct values refer S1 Table.

Similarly, 93 pools contained samples that tested negative on individualized RT-PCR and 92 of these pools were also found to be negative on pooled analysis. One of the 93 pools tested positive on pooled analysis with a Ct value of 33.3; though on deconvolution the individual samples tested negative.

The prevalence-dependent and prevalence-independent diagnostic characteristics of the pooled sample analysis strategy are depicted in Table 2 (this table in a different format is included as S2 Table), with PPV and NPV simulated at 1% to 5% background positivity. We observed 75.0% (47.6% to 92.7%) sensitivity, 98.9% (94.2 to 100%) specificity, 95.8% (90.8% to 98.2%) NPV and 0.3 (0.1–0.6) Negative Likelihood Ratio values for pooled analysis. The extent of agreement between pooled versus individual sample strategy was estimated further by kappa statistic (κ = 0.8; 95% CI = 0.6–1.).

**Table 2:** Diagnostic characteristics of the pooled sample analysis strategy.

A. Prevalence independent parameters			B. Prevalence dependent parameters			
Statistic	Value	95% CI	Assumed Prevalence	Statistic	Value	95% CI
Sensitivity	75.0%	47.6 to 92.7%	1%	PPV	41.3%	9% to 83.5%
Specificity	98.9%	94.2% to 100%		NPV	99.8%	99.4% to 99.9%
Positive Likelihood Ratio	69.8	9.7 to 500.1	2%	PPV	58.7%	16.6% to 91.1%
Negative Likelihood Ratio	0.3	0.1 to 0.6		NPV	99.5%	98.8% to 99.9%
Kappa statistics	0.8 ± 0.1	0.6 to 1.	3%	PPV	68.3%	23.1% to 93.9%
				NPV	99.2%	98.2% to 99.7%
	Agreements by chance: 85.7 (77.2%)		4%	PPV	74.40%	28.8% to 95.4%
				NPV	99%	97.6% to 99.6%
	Observed agreements: 106 (95.5%)		5%	PPV	78.6%	33.7% to 96.3%
				NPV	98.7%	97% to 99.4%

Assuming PCR-positivity to reflect disease prevalence in the community, for point prevalence of 4.8% (26/545):

PPV = 92.3% (62.6% to 98.6%)

NPV = 95.8% (90.8% to 98.2%)

The pooled strategy expectedly led to a significant gain in turn-around time. The total laboratory processing time required for the analysis of 545 samples through individualized sampling strategy was 67 hours, while the same was found to be 23 hours for pooled sampling including the testing of individual samples after deconvolution of the positive pools. Similarly, the pooled testing strategy led to 68% savings in reagent costs with the total cost of individualized testing being $3137 whereas the pooled analysis including the deconvoluted samples was $1002.

## Discussion

In this study we report that the pooled sample analysis strategy, if applied in the scenario of an emerging outbreak of COVID-19, offers a significant reduction of laboratory turn-around time and reagent requirement at the cost of compromised diagnostic sensitivity. At the reported point prevalence of 4.8% among the individuals tested in this study, the NPV of pooled sample strategy was around 96%; thereby suggesting the possibility of missing infected cases and risking community transmission from undiagnosed individuals. Particularly, presence of a single infected individual with relatively low viral load (Ct value of ≥ 34), in a pool of 5, was likely to be missed in pooled sample analysis. One of the reasons for this could be the dilution of sample beyond the limit of detection of the assay used.

Since PCR-positivity is a function of the net viral load in the pooled sample, we understand that the success of the pooling strategy as a sensitive screening tool would be dependent on the number of infected patients in the pool and the viral load in the individual samples. While the latter is likely to be influenced by host-specific factors like immune competence, comorbidities and age, the former is likely to be a reflection of the background prevalence of infection in the community. While pooled sample testing offers the advantage of cost effectiveness and timely reporting, the strategy becomes less useful in communities where the prevalence of the disease is high. With increased prevalence, number of positive pools are likely to increase and more pools are to be deconvoluted for identifying the positive individuals negating the advantages such as cost and time saving. Furthermore, with the increase in the prevalence of a disease, NPV of the diagnostic test for the disease decreases leading to higher false negative results. Based on this premise, national advisories have suggested testing of 5 sample pools for COVID-19. in communities with prevalence of upto 5% [6]. However, our data reflects the lack of a robust relationship between the former factor and the Ct value of the pooled sample; thus, implying the greater role of viral titers in individual samples in influencing the PCR results. The relatively low sensitivity and resultant negative predictive value of the pooled sample analysis strategy hint at its weakness as an effective screening tool in reliably “ruling out” the diagnosis of COVID-19. In our study, pooled sampling led to false negative results in 4 of the 545 patients; all of whom were direct household contacts of known COVID-19 patients and two of them were clinically symptomatic with cough, sore throat and fever. Despite being clinically and epidemiologically suggestive, three of these 4 patients had Ct values above 34; thereby suggesting that pooled analysis is likely to miss the detection of samples with low viral loads. These instances of missed case detection pose the risk of community transmission of infection from undetected sources.

Earlier studies evaluating the utility of pooled sample testing for surveillance of bovine viral diarrhea virus and porcine reproductive and respiratory syndrome virus demonstrated that sensitivity of RT-PCR tests reduced when run on pooled samples, due to a dilution effect [7, 8]. In a recently published study Lohse et al., demonstrated that pooling of up to 30 samples could detect E gene and S gene and difference between Ct values of individual positive sample and pooled sample was up to 5. One of the reasons cited by the authors for not missing a positive individual sample in the pooled assay could be the high viral titers of the positive individual samples as the Ct of individual samples ranged from 18 to 30. Individual samples with Ct value of 34 were detected as positive in this study as a cut-off value of 45 was used by the authors [9].

Of the 93 pools created from samples testing negative on individualized testing, 92 were negative on pooled analysis and one pool tested positive with Ct of 33.3 for *Orf1ab* gene. However, on deconvolution, each of the 5 constituent samples in that pool tested negative. We observed that this pool comprised of 3 patients with severe acute respiratory infection. We surmise that non-specific inhibitors of PCR amplification, co-habiting the viral genomic target in any of these patients, might have suppressed the amplification of low-level viral target in individual samples beyond the detectable threshold. Pooling the samples could potentially dilute these inhibitors and lead to a detectable signal with a relatively delayed Ct on poled analysis. In a recent study Hogan and co-workers made a similar observation while assessing the utility of pooled sample testing strategy to detect community transmission of SARS-CoV-2 in San Francisco Bay Area, CA, USA. One of the pools was positive in their study, however, upon deconvolution all the individual sample tested negative [10].

Our study suffered from several limitations. Apart from having a relatively small sample size, as mentioned above, the 3 districts did not have similar background prevalence. Hence the diagnostic characteristics like positive and negative predictive values are likely to be dissimilar between the districts. Furthermore, pooling of consecutive samples was done in the study. While family members of infected cases were likely to be included in the same pool which may have decreased the possibility of false negative results. adopting a strategy of random pooling could lead to a different proportion of infected individuals in the pools, the effect of which was not explored in the study. We also did not have pools in which multiple infected samples had Ct values of ≥ 34 and, hence, the diagnostic performance of such pools could not be assessed.

We therefore, conclude that though the pooling strategy could be an alternative in resource-constrained settings with overwhelmed laboratory infrastructure, its adoption could miss the detection of pools having single infected individuals with low viral loads. Our study, which is probably the first report on pooled testing in areas experiencing the early phase of emergence of COVID-19 outbreaks, shows that 15.4% of the infected pools could be missed by this strategy. It is therefore recommended that adoption of the pooled sample testing strategy, as a cost effective screening tool for early containment of transmission in emerging outbreaks, has to be tailored to the prevalence of COVID-19 in the geographical area concerned.

## Supporting information

S1 Table Ct values of individual and pooled samples. (XLSX)

S2 Table Diagnostic characteristics of the pooled sample analysis strategy. (DOCX)
