# Supplementary material for: Evaluation of pooled sample analysis strategy in expediting case detection in areas with emerging outbreaks of COVID-19: A pilot study
Source: PLoS One. 2020 Sep 22;15(9):e0239492. doi: 10.1371/journal.pone.0239492 (PMC7508355; doi:10.1371/journal.pone.0239492)
Supplement: S2 Table — (DOCX) [file pone.0239492.s002.docx]

**Table S2 Diagnostic characteristics of the pooled sample analysis strategy.**

| 1. **Prevalence independent parameters** | | | | | | | | | | | | | | |
| --- | --- | --- | --- | --- | --- | --- | --- | --- | --- | --- | --- | --- | --- | --- |
| **Statistics** | **Sensitivity (%)** | | | | **Specificity (%)** | | | **Positive Likelihood ratio** | | | **Negative likelihood ratio** | | | |
| Value | 75.0 | | | | 98.9 | | | 69.8 | | | 0.3 | | | |
| 95% CI | 47.6 to 92.7 | | | | 94.2 to 100.0 | | | 9.7 to 500.1 | | | 0.1 to 0.6 | | | |
| **Kappa statistics** | | | | | | | | | | | | | | |
| Value | 0.8 ± 0.1 | | | | | | | | | | | | | |
| 95% CI | 0.6 to 1.0 | | | | | | | | | | | | | |
| Agreement by chance | 85.7 (77.2%) | | | | | | | | | | | | | |
| Observed agreements | 106 (95.5%) | | | | | | | | | | | | | |
| 1. **Prevalence dependent parameters** | | | | | | | | | | | | | | |
| Assumed prevalence (%) | | 1 | | 2 | | | 3 | | | 4 | | | 5 | |
| **Statistics** | | **PPV** | **NPV** | **PPV** | | **NPV** | **PPV** | | **NPV** | **PPV** | | **NPV** | **PPV** | **NPV** |
| Value (%) | | 41.3 | 99.8 | 58.7 | | 99.5 | 68.3 | | 99.2 | 74.4 | | 99 | 78.6 | 98.7 |
| 95% CI (%) | | 9 to 83.5 | 99.4 to 99.9 | 16.6 to 91.1 | | 98.8 to 99.9 | 23.1 to 93.9 | | 98.2 to 99.7 | 28.8 to 95.4 | | 97.6 to 99.6 | 33.7 to 96.3 | 97 to 99.4 |
| Assuming PCR-positivity to reflect disease prevalence in the community, for point prevalence of 4.8% (26/545):  PPV = 92.3% (62.6% to 98.6%)  NPV = 95.8% (90.8% to 98.2%) | | | | | | | | | | | | | | |
